# Evolutionary patterns and future perspectives of joint replacement in arthritis patients: a comprehensive analysis of findings over the past decades

**DOI:** 10.1530/EOR-2025-0071

**Published:** 2025-09-04

**Authors:** Sha-Sha Tao, Jian Tang, Yu-Chen Liu, Shu-Zhen Xu, Zhu Chen, Hai-Feng Pan

**Affiliations:** ^1^ Department of Epidemiology and Biostatistics, School of Public Health, Anhui Medical University, Hefei, Anhui, China; ^2^ Inflammation and Immune Mediated Diseases Laboratory of Anhui Province, Hefei, Anhui, China; ^3^ Preventive Medicine Experimental Teaching Center, School of Public Health, Anhui Medical University, Hefei, Anhui, China; ^4^ Department of Otolaryngology, Head and Neck Surgery, The First Affiliated Hospital of Anhui Medical University, Hefei, Anhui, China; ^5^ Department of Rheumatology and Immunology, The First Affiliated Hospital of USTC, University of Science and Technology of China, Hefei, Anhui, China

**Keywords:** joint replacement, arthritis, evolutionary patterns, future perspectives, patient-centered care

## Abstract

**Purpose:**

- This study aimed to comprehensively review the current research status and trends of joint replacement for arthritis patients worldwide.

**Methods:**

- Literature related to joint replacement for arthritis patients from 2004 to 2024 was extracted from the Web of Science Core Collection (WoSCC) database. A systematic qualitative and quantitative analysis of these publications was conducted. Visualization of results was achieved using CiteSpace and VOSviewer software.

**Results:**

- In total, 14,349 publications met the inclusion and exclusion criteria and were selected for further research. Keyword analysis revealed a clinical emphasis on optimizing surgical outcomes through advancements in total knee arthroplasty, total hip arthroplasty, and patient-centered metrics. Emerging research hotspots since 2020 included precision techniques such as robotic-assisted surgery, strategies to address periprosthetic joint infection, and the role of psychological factors such as depression and patient satisfaction. Temporal trends highlighted evolving priorities, including personalized alignment protocols, where the burst intensities of kinematic alignment and mechanical alignment were 16.33 and 13.79, respectively, as well as functional recovery and complication prevention. Historical dominance of rheumatoid arthritis research has transitioned to broader exploration of osteoarthritis management and technological innovations in surgical precision.

**Conclusion:**

- This study revealed a marked transition from rheumatoid arthritis-focused studies to innovations in osteoarthritis management and precision surgical techniques. Clinically, these findings emphasize the need to integrate technological advancements with multidisciplinary frameworks to standardize infection control, optimize implant durability, and enhance rehabilitation outcomes. Future efforts should prioritize personalized alignment strategies, evidence-based infection control protocols, and multidisciplinary rehabilitation frameworks to improve long-term functional outcomes.

## Introduction

Arthritis is an inflammatory disease of the joints and surrounding tissues caused by factors such as inflammation, infection, degeneration, trauma, or other causes. It is characterized by joint redness, swelling, heat, pain, functional impairment, and joint deformities (1). Severe cases of arthritis can lead to joint dysfunction, impacting the quality of life and mental health of patients (2, 3, 4, 5). There are over 100 recognized types of arthritis (1). Among them, osteoarthritis (OA), rheumatoid arthritis (RA), and gout are particularly common, and these three types of arthritis have high prevalence and disability risks globally. They not only bring immense suffering to patients but also constitute a heavy disease burden, making them a significant issue requiring urgent attention in the field of public health (5, 6, 7, 8).

Joint replacement surgery is a common treatment method used to address severe arthritis and is widely recognized as the most effective treatment for advanced arthritis, significantly improving outcomes for arthritis patients (9). However, joint replacement surgery also carries certain risks and limitations, including surgical indications, surgical complications, postoperative recovery processes, and the lifespan of artificial joints (10, 11, 12). In addition, factors such as the patient’s age, race, health status, and lifestyle also influence the surgical outcome (13, 14, 15). A comprehensive study of the relevant literature on joint replacement surgery and arthritis patients, understanding the practical application effects of joint replacement surgery in different types of arthritis patients, staying up to date with the latest research findings on joint replacement surgery, and mastering the latest surgical techniques and materials can help researchers determine the optimal surgical timing, refine procedural approaches, and optimize postoperative rehabilitation strategies. This contributes to improving the quality of treatment, reducing the occurrence of complications, and enhancing the quality of life for patients.

As time progresses and disciplinary research deepens, the field of joint replacement in arthritis patients has accumulated a vast amount of scientific papers, providing researchers with a wealth of literature. However, researchers also face multiple challenges when exploring this field. First, the diversity of the literature poses a major challenge. The topics are wide-ranging, the research focuses vary, types of studies are diverse, and their depths vary, with varying quality, making it difficult for researchers to quickly locate key information during screening and reading. Second, the publication patterns of journals vary, with different journals having different publication types and requirements, which increases the difficulty for researchers in selecting and evaluating the literature. Furthermore, faced with the vast number of published articles, many of which may contain similar content, efficiently evaluating and selecting the target literature becomes a problem that researchers must address.

To assist novice researchers in quickly and systematically mastering the professional knowledge in the field of joint replacement for arthritis patients, it is necessary to comprehensively review and integrate the literature in this field. Bibliometric analysis is a kind of literature analysis method that can quantitatively and qualitatively analyze the quantity, publication patterns, and publication characteristics of the published literature in a particular field (16). Bibliometric studies have already been used in various aspects for systematically assessing publications in a certain field of study (17, 18). It can analyze the author, keywords, journals, countries, institution, references, and other information of publications, using visualization tools, including CiteSpace, VOSviewer, and HistCite tools, so as to provide clues and theoretical basis for studying the research trends and focus of various disciplines (17, 19). This not only helps researchers gain in-depth understanding of the basic theories, cutting-edge developments, hot topics, and challenges in the field, but also provides them with an overview of journal contents, thematic developments, and influencing factors, thereby accelerating their learning process.

Therefore, this study aimed to synthesize global research trends in joint replacement for arthritis to address critical clinical challenges, including surgical precision, implant longevity, and post-operative complications such as periprosthetic joint infection (PJI). This literature analysis provides actionable insights to optimize surgical outcomes, refine patient selection criteria, and guide arthritis management. These findings directly inform clinical practice by bridging gaps between technological advancements and patient-centered care, ultimately enhancing functional recovery and long-term quality of life for individuals undergoing joint replacement.

## Methods

### Data processing

**Figure 1:**
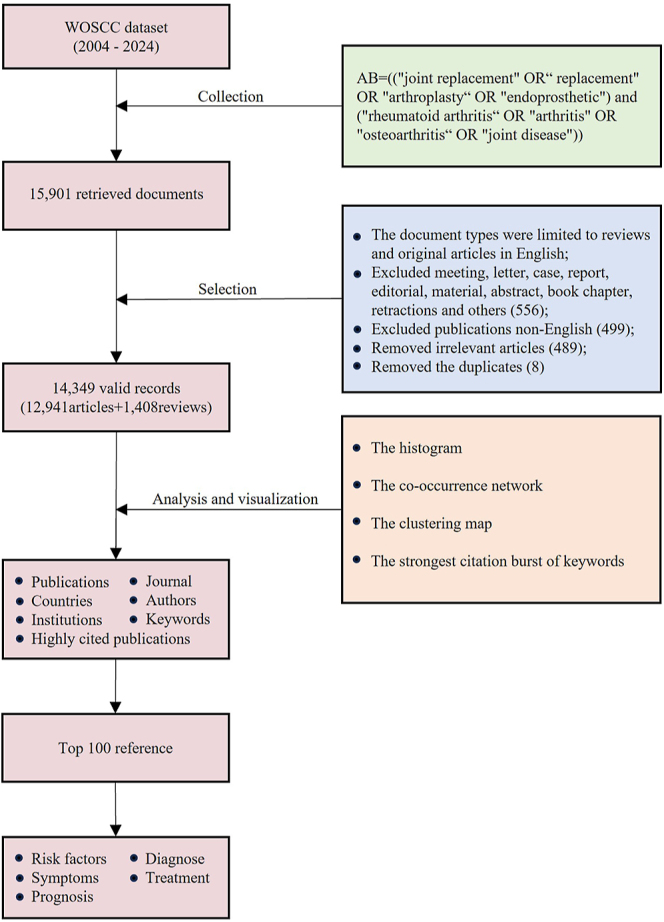
Flowchart of the literature selection.

Web of Science (WOS), one of the world’s most trusted publishers of global citation databases, is a leading platform for scientific research (20). The literature was identified by searching the WOS Core Collection (WOSCC) database using the detailed search strategy as follows: AB = ((‘joint replacement’ OR ‘replacement’ OR ‘arthroplasty’ OR ‘endoprosthetic’) and (‘RA’ OR ‘arthritis’ OR ‘osteoarthritis’ OR ‘joint disease’)). Because WOSCC only covers the literature published in the last 20 years, the literature we analyzed was from January 1, 2004 to June 4, 2024. A total of 15,901 publications were researched. A hierarchical filtering process was used to ensure all study types were mutually exclusive and that each article was only indexed for one study type. We reviewed a random sample of 1,000 records to ensure classification accuracy for specialty and study type. The document types were limited to review articles (systematic or otherwise) and original articles (any design) in English. After removing meeting abstracts, letters, case reports, editorials, book chapters, abstracts, and retractions, 15,345 publications were included. After excluding 499 non-English publications, 14,846 publications were included. By reading abstracts, 489 irrelevant articles were manually removed. After removing eight duplicate publications, a total of 14,349 publications, including 12,941 articles and 1,408 reviews, were selected as the final data set for further analysis. The search and analysis processes are shown in Fig. 1. We conducted a detailed statistical description of the selected literature, including types of documents, most prolific articles, authors, institutions, and countries. In our study, ethical review was not required (Fig. 1).

### Statistical analysis

The screened 14,394 data were used for analyzing information including publication year, quantity, authors, institutions, countries, citation counts, keywords, and their interactive relationships by Microsoft Office Excel 2023, CiteSpace (v.6.2.R4 Advanced) (21), and VOSviewer (v.1.6.19) (22). The histogram generated by Microsoft Office Excel visualized the distribution of publications across different years, countries, research institutions, and authors. CiteSpace and VOSviewer were used to create co-occurrence networks, in which the size and color of the circles represent the number and year of publications, respectively, and the number and thickness of the lines between the circles represent the frequency and intensity of cooperation between the researchers, respectively (23). The clustering map and timeline map were drawn by CiteSpace according to the logarithmic likelihood ratio of keywords and were used to describe the research direction and the progress of research hotspots over time. When the clustering modular value Q (modularity Q) >0.3 and the clustering contour index S (mean silhouette) >0.5, this indicates a reasonable clustering with a clear structure and high reliability. The clustering map and timeline map in this study all met the requirements. CiteSpace software was used to analyze the strongest citation burst of keywords, which could describe the frequency, intensity, and time period of high-frequency keywords and indicate the research hotspots in the field (24, 25). The study has been reported in line with the STROCSS criteria (26). The review process was conducted according to the Preferred Reporting Items for Systematic Reviews and Meta-Analyses (PRISMA) guidelines.

## Results

### Publication numbers sorted by year

**Figure 2:**
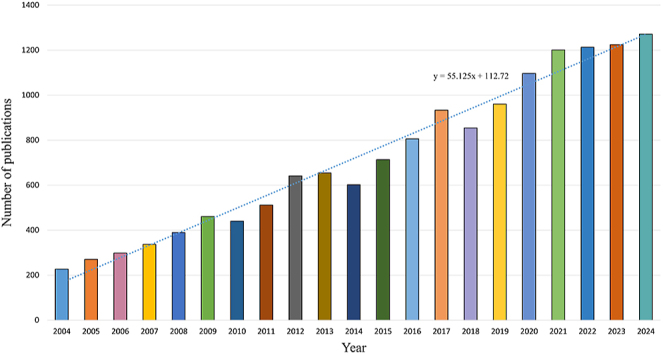
Annual distribution of publications on joint replacement for arthritis patients from 2004 to 2024.

In total, 14,349 publications including 12,941 (90.19%) articles and 1,408 (9.81%) reviews, were analyzed using the search method. Over the past two decades, the annual publication distribution has exhibited a generally consistent upward trend with fluctuations. In particular, there were significant increases in the number of publications in 2009, 2012, 2013, 2017, 2020, and 2021. In addition, the rate of increase after 2014 was generally higher compared to before 2014. From 2014 onward, the number of publications remained relatively stable at around 1,200 annually, with a slight increase observed from 2021 to 2023 (Fig. 2).

### Country distribution of publications

#### Country co-occurrence network

The 14,349 publications on joint replacement for arthritis patients came from 108 different countries and were unevenly distributed. The United States (US) stood out significantly with a leading number of 4,382 publications. Following closely were the United Kingdom (UK), China, Japan, and Germany, with 1,523, 1,401, 1,072, and 955 publications, respectively. Centrality can reflect the academic influence of countries, and nodes with a centrality greater than 0.1 are considered to be important. In terms of centrality, the top five countries were the US (0.24), France (0.16), Spain (0.15), the UK (0.13), and Belgium (0.12), while the centrality of other countries was below 0.1.

#### Country clustering map

**Figure 3:**
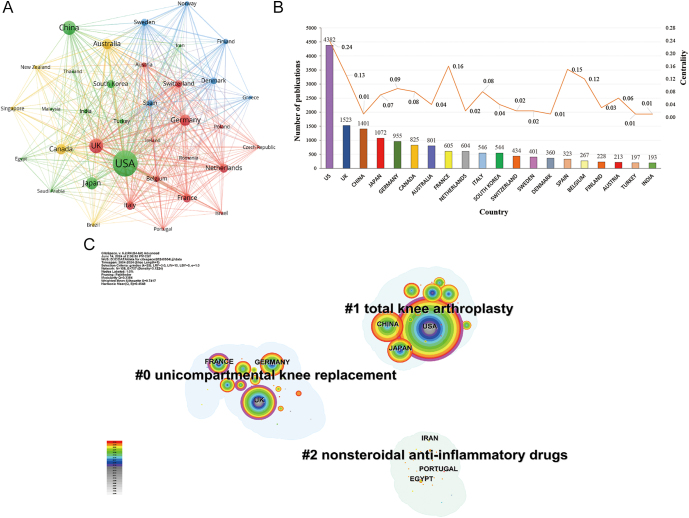
The country distribution of publications on joint replacement for arthritis patients. (A) the co-occurrence network of countries; (B) top 20 countries by number of publications and their centrality; (C) the clustering map of countries.

The country clustering map categorizes research hotspots of various countries into three main categories: #0 ‘unicompartmental knee replacement (UKR)’, #1 ‘total knee arthroplasty (TKA)’, and #2 ‘non-steroidal anti-inflammatory drugs (NSAIDs)’ (Fig. 3). The UK, Germany, and France primarily focused on the field of UKR. The UK began to focus on this area from 2006, with peak interest observed from 2019 to 2021. Germany has shown steady and increasing interest in this field since 2015, maintaining significant activity through 2024. France emerged prominently in research on this topic from 2014 and maintains high centrality, with strong connections to other countries in this domain. Meanwhile, countries such as the US, China, and Japan delved deeper into research on TKA. The US has shown sustained interest over a longer period, starting from 2004. China’s interest has steadily risen since 2017, with consistent peaks in recent years. Japan’s focus on this area began in 2015, spiked in 2017, and has since maintained a stable level of activity. Countries such as Egypt, Iran, and Portugal primarily concentrate on the use of NSAIDs. Interest in this area began notably after 2021, marking it as a newer research hotspot.

### Institution distribution of publications

#### Institution co-occurrence network

**Figure 4:**
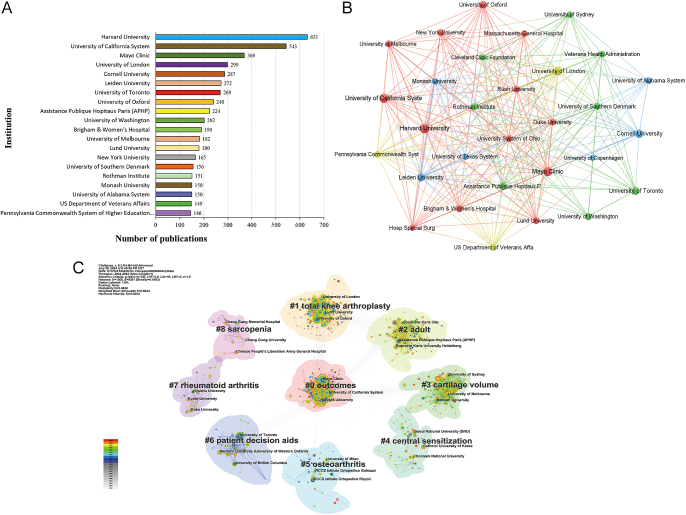
The institution distribution of publications on joint replacement for arthritis patients. (A) top 20 institutions by number of publications; (B) the co-occurrence network of institutions; (C) the clustering map of institutions.

According to a detailed analysis of the institution co-occurrence network, we identified a total of 555 publishing institutions (Fig. 4). Among them, Harvard University, the University of California System, and the Mayo Clinic ranked in the top three, with 631, 543, and 369 publications, respectively. It is worth noting that all these three institutions are located in the US. Among the top 20 institutions by publication number, 11 institutions are based in the US. These 11 institutions collectively contributed to 68.07% of the total publications of the US, which undoubtedly contributed to the significant lead of the US in terms of publication output. In addition, among the remaining nine institutions in the top 20, Australia and the UK each had two, while the Netherlands, Canada, France, Sweden, and Denmark each had one institution. It is noteworthy that despite China, Japan, and Germany ranking in the top five for publication numbers, none of their institutions were among the top 20. This could be due to the relatively dispersed and evenly distributed publication numbers across institutions within these countries.

#### Institution clustering map

Through clustering analysis of research directions among institutions, nine categories were identified: #0 ‘outcomes’, #1 ‘TKA’, #2 ‘adult’, #3 ‘cartilage volume’, #4 ‘central sensitization’, #5 ‘osteoarthritis’, #6 ‘patient decision aids’, #7 ‘RA’, and #8 ‘sarcopenia’ (Fig. 4). Among the top ten ranked institutions, there was a significant focus on research related to #0 ‘outcomes’ and #1 ‘TKA’. Specifically, #0 ‘outcomes’ attracted four institutions within the top ten rankings, including Harvard University (ranked 1st), the University of California System (2nd), the Mayo Clinic (3rd), and Cornell University (6th). Meanwhile, #1 ‘TKA’ attracted the University of London (ranked 4th), Cornell University (5th), and the University of Oxford (8th).

### Journal analysis

**Figure 5:**
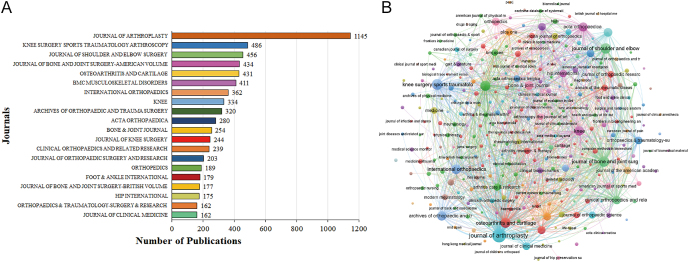
The journal distribution of publications on joint replacement for arthritis patients. (A) top 20 journals by number of publications; (B) the co-occurrence network of journals.

We analyzed journal data sources, with the ‘Journal of Arthroplasty’ leading significantly, publishing an astonishing 1,145 publications. Figure 5 displays the top 20 journals in the field of joint replacement, along with the number of articles published in each. We categorized the top 20 journals into four main groups: journals focused on joint replacement and arthroscopic surgery, such as ‘Knee Surgery, Sports Traumatology, Arthroscopy’, ‘Journal of Shoulder and Elbow Surgery’ and ‘Journal of Bone and Joint Surgery-American Volume’; journals specializing in osteoarthritis and cartilage research, such as ‘Osteoarthritis and Cartilage’; journals covering bone, joint, muscle disorders, and surgical research, including ‘BMC Musculoskeletal Disorders’ and ‘International Orthopaedics’; journals in the field of foot, ankle, and other orthopedic specialties, such as ‘Foot & Ankle International’, ‘Journal of Bone and Joint Surgery-British Volume’, ‘HIP International’, and ‘Orthopaedics & Traumatology: Surgery & Research’; and interdisciplinary journals such as ‘Journal of Clinical Medicine’.

### Author distribution of publications

#### Author co-occurrence network

**Figure 6:**
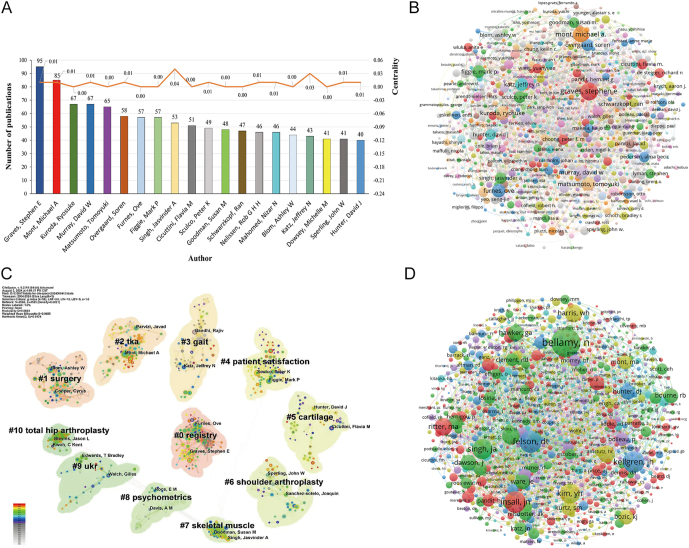
The author distribution of publications on joint replacement for arthritis patients. (A) top 20 authors by number of publications and their centrality; (B) the co-occurrence network of authors; (C) the clustering map of authors; (D) the co-cited network of authors.

In the detailed analysis of the co-linearity chart in Fig. 6, we focused particularly on two authors who have made outstanding contributions in their respective fields: Stephen E Graves and Michael A Mont. These scholars have achieved remarkable publication counts of 95 and 85 papers, respectively, placing them significantly ahead of other researchers. Stephen E Graves stands out with his exceptional 95 research publications, establishing himself as a prominent figure in academia. As a member of the Australian Orthopaedic Association National Joint Replacement Registry, while the organization ranks 37th in total publications, Graves’ personal contributions astonishingly account for 87.16% of the organization’s total publications in this field. This clearly demonstrates his academic leadership and profound influence. Following closely behind Graves, Michael A Mont has similarly garnered widespread attention for his outstanding performance. Michael A Mont is a distinguished expert in the field of orthopedics, particularly renowned for his expertise in joint preservation and reconstruction. He is affiliated with the Rubin Institute for Advanced Orthopedics, located at Sinai Hospital of Baltimore, which is a leading center dedicated to advanced orthopedic research and clinical practice.

#### Author clustering map

Cluster analysis (Fig. 6) categorized authors into 11 groups according to their research directions: #0 ‘registry’, #1 ‘surgery’, #2 ‘TKA’, #3 ‘gait’, #4 ‘patient satisfaction’, #5 ‘cartilage’, #6 ‘shoulder arthroplasty’, #7 ‘skeletal muscle’, #8 ‘psychometrics’, #9 ‘UKR’, and #10 ‘total hip arthroplasty’.

#### Co-cited author analysis

Co-authorship analysis serves as a powerful tool, providing an in-depth understanding of the complex network of connections among authors, institutions, and countries within a specific research field or journal. The results of co-citation analysis showed that the top five most cited authors were Nicholas A Bellamy, David T Felson, John N Insall, JH Kellgren, and Young Ho Kim, with cumulative citation counts of 1,554, 1,084, 1,041, 1,034, and 959, respectively (Fig. 6).

### Keyword analysis

#### Keywords co-occurrence network

**Figure 7:**
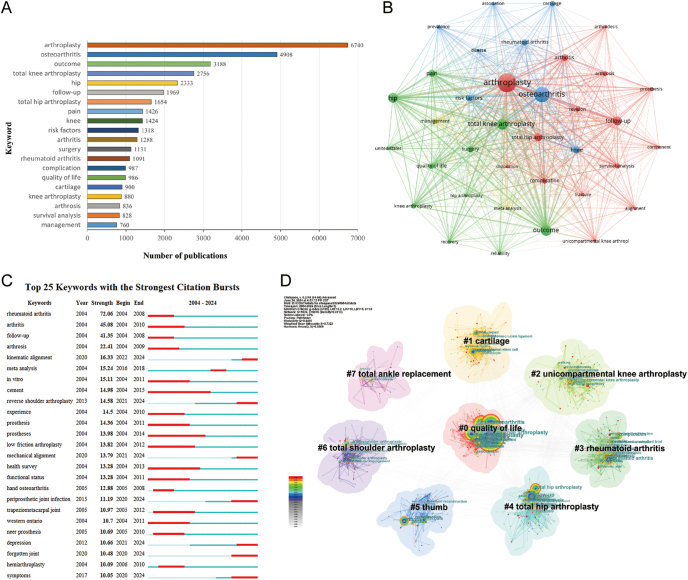
The keyword distribution of publications on joint replacement for arthritis patients. (A) top 20 keywords by number of publications; (B) the co-occurrence network of keywords; (C) the top 25 keywords with the strongest citation bursts; (D) the clustering map of keywords.

From 14,349 publications, keywords were extracted and semantically similar ones were merged, resulting in a final set of 1,000 keywords. These keywords form a co-occurrence network (as shown in Fig. 7), where the size of each node reflects the frequency of the keyword's appearance in the literature. Keywords from different years are marked with different colors to highlight their occurrence over time. Among these keywords, 13 appeared more than 1,000 times each. These include ‘arthroplasty’, ‘osteoarthritis’, ‘outcome’, ‘TKA’, ‘hip’, ‘follow-up’, ‘total hip arthroplasty’, ‘pain’, ‘knee’, ‘risk factors’, ‘arthritis’, ‘surgery’, and ‘RA’. Their publication frequencies were 6,740, 4,908, 3,188, 2,756, 2,333, 1,969, 1,654, 1,426, 1,424, 1,318, 1,288, 1,131, and 1,091 publications, respectively.

#### Keywords clustering map

**Figure 8:**
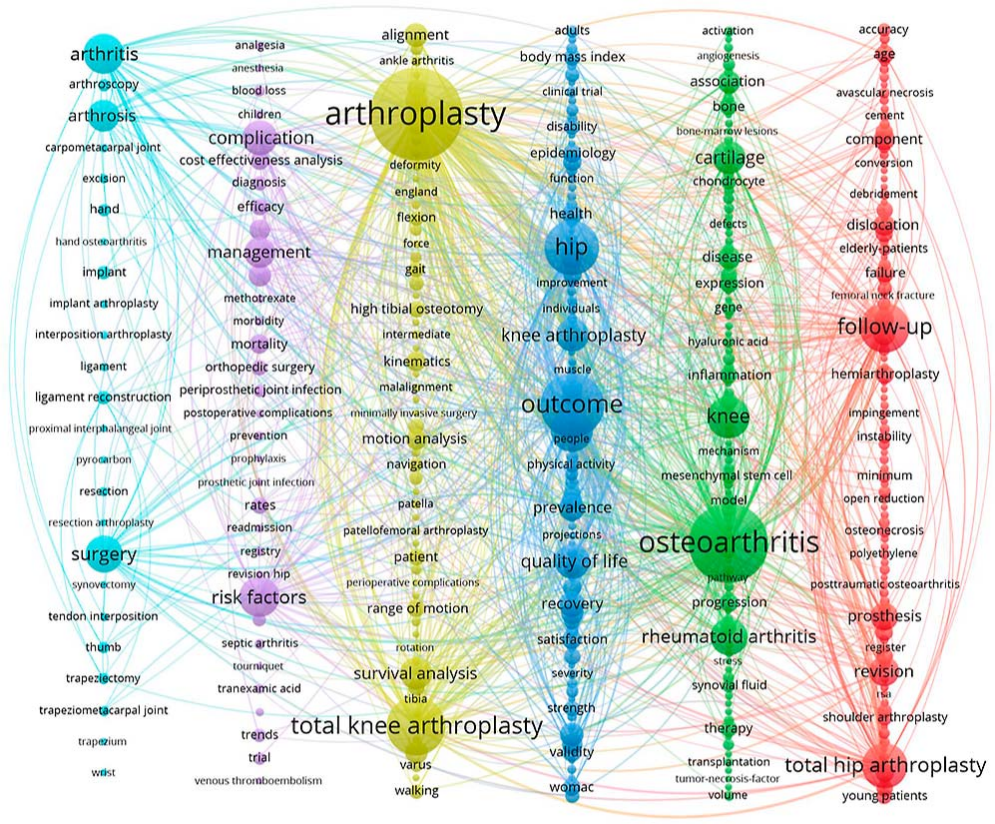
Keyword co-occurrence network line map of publications on joint replacement in arthritis patients.

As shown in Fig. 7, the keywords were categorized into eight core categories: #0 ‘quality of life’; #1 ‘cartilage’; #2 ‘unicompartmental knee arthroplasty’; #3 ‘RA’; #4 ‘total hip arthroplasty’; #5 ‘thumb’; #6 ‘total shoulder arthroplasty’; and #7 ‘total ankle replacement’. This classification system comprehensively and systematically covers the main domains of the keywords. As shown in Fig. 8, the keyword clustering map provides an overview of the key terms and their relationships in the field of arthroplasty research. For example, the green cluster centers on ‘osteoarthritis’ and includes ‘knee’ and ‘RA’, highlighting the focus on joint replacement for these conditions. The blue cluster emphasizes outcomes, with keywords such as ‘outcome’, ‘quality of life’, and ‘recovery’.

#### Keywords with strongest citation bursts

Using burst analysis allows the precise capture of keywords that have experienced a significant surge in frequency within specific time periods. This approach facilitates the evaluation of the present focus in this field and reflects the future research development pattern. Figure 7 presents the results of this analysis, displaying the top 25 keywords by burst intensity. Leading the list is ‘RA’, with a significant burst intensity of 72.06, indicating sustained widespread attention from 2004 to 2008, making it a focal point in the field. Following closely are ‘arthritis’ and ‘follow-up’ with burst intensities of 45.08 and 41.35, respectively, occupying the second and third positions. Of particular note, Fig. 7 also reveals a set of keywords that have surged in popularity since 2020, including ‘kinematic alignment’, ‘reverse shoulder arthroplasty’, ‘mechanical alignment’, ‘PJI’, ‘depression’, ‘forgotten joint’, and ‘symptoms’. These keywords have burst intensities of 16.33, 14.58, 13.79, 11.19, 10.66, 10.48, and 10.05 respectively, indicating these areas are becoming new research hotspots and garnering public attention. This discovery not only reflects the dynamic shifts in research trends but also provides crucial insights for navigating future development directions.

### Analysis of top 100 cited publications

To further elucidate clinical research priorities and emerging trends in joint replacement for arthritis, we analyzed the top 100 most-cited publications identified from the dataset (Supplementary Table 1, (see section on Supplementary materials given at the end of the article)). Each article was meticulously examined and categorized by its primary focus. Together, these papers present a comprehensive overview of the fields of arthritis and joint replacement surgery. These papers extensively cover multiple dimensions, ranging from disease management strategies to surgical technological innovations, as well as patient quality of life assessments and epidemiological trend analyses. They deeply reflect the cutting-edge dynamics of current clinical practice and research.

These articles were categorized into five main areas: risk factors, diagnosis, symptoms, treatment, and prognosis. Current research hotspots primarily focus on understanding and managing risk factors, improving diagnostic accuracy, and optimizing treatment outcomes. Diagnostic tools and criteria, including the classification of osteoarthritis, radiographic evaluation, and clinical scoring systems, are crucial for accurate diagnosis and treatment planning.

Treatment modalities, ranging from cemented and uncemented total hip replacements to total knee and shoulder arthroplasties, were a major focus. Non-surgical management of knee osteoarthritis and cost-benefit analyses of various treatments were also highlighted, reflecting the need for comprehensive and cost-effective care. The field is expected to emphasize personalized surgical approaches, leveraging advancements in imaging and genetic profiling to tailor treatments to individual patient needs. In addition, there will be a continued emphasis on improving post-operative outcomes, including pain management, quality of life, and functional improvement, as well as minimizing complications and enhancing patient satisfaction.

## Discussion

Joint replacement surgery is one of the effective treatment methods for patients with arthritis and has attracted the attention of many clinical practitioners and epidemiological experts. Our bibliometric analysis reveals the complex and evolving publication trends of the literature related to joint replacement surgery: from optimizing traditional arthroplasty techniques to integrating precision innovations (e.g., robotic systems) and prioritizing patient-centric outcomes. This progression suggests the field’s transition from procedural refinement to holistic care models that focus on long-term mobility and psychosocial well-being.

Annual publication trends provide valuable insights into the rate and advancement of our research. Temporal analysis of 14,349 publications reveals sustained growth in joint replacement research, with accelerated output after 2014 and peaks in 2012, 2017, and 2020. These surges correlate with milestones such as the adoption of robotic-assisted surgery and PJI management guidelines. The trajectory suggests an increasing emphasis on translating technological advancements into clinical protocols to address the rising prevalence of osteoarthritis and aging populations. It can be predicted that a large number of researchers will continue to delve deeper into this field in the future.

Geographic analysis reveals that the US, UK, and China are leading centers of research output, with the US dominating both the number of publications and collaboration centrality. Within the US, institutions such as Harvard University (27), the University of California system (28), and the Mayo Clinic (29) not only demonstrate high research productivity but also maintain extensive internal collaborations and partnerships with other research entities. However, despite this strong domestic connectivity, global collaboration remains limited. In addition, the publication analysis of countries such as China, Germany, and Japan highlights a similar challenge: insufficient international cooperation.

In the journals associated with the literature in this study, we identified that the top 20 journals by publication count can generally be categorized into four main types. First, journals centered on joint replacement and arthroscopic surgery mainly cover topics such as joint replacement procedures, arthroscopic techniques, and related shoulder and elbow orthopedic treatments, including disease management, surgical methods, and clinical studies, exemplified by the ‘Journal of Arthroplasty’ (30). Second, journals focusing on OA and cartilage research are committed to exploring areas related to OA and cartilage, investigating disease mechanisms, therapeutic approaches, and clinical applications, as seen in ‘Osteoarthritis Cartilage’ (31). Third, journals dedicated to musculoskeletal diseases and surgical research address a variety of musculoskeletal system disorders, injuries, and surgical treatments, encompassing clinical research, surgical techniques, and treatment strategies, such as ‘BMC Musculoskeletal Disorders’ (32). Fourth, journals in the field of foot-ankle and other orthopedic areas concentrate on different aspects of foot-ankle conditions, hip joint diseases, and orthopedic surgical research, thoroughly examining clinical practices, surgical techniques, and treatment innovations, such as ‘Foot and Ankle Surgery’ (33). In addition, interdisciplinary journals span beyond orthopedics, covering a wide range of medical fields, discussing various clinical practices, treatment methods, and disease management strategies, such as ‘International Journal of Surgery’ (34). Each category of journal offers in-depth academic research, extensive clinical experience sharing, and the latest technological advancements in their respective fields, providing valuable information resources and a platform for researcher collaboration.

We conducted a detailed analysis of the top 30 most-cited articles in the field of arthritis and joint replacement surgery. These articles can be summarized into four main aspects: disease management and treatment recommendations, quality of life and patient satisfaction, epidemiology and prognosis studies, and biomedical engineering and therapeutic technologies. In terms of disease management and treatment recommendations, evidence-based expert consensus guidelines, such as those from OARSI (35) and EULAR (36) for hip and knee OA, respectively, offer strong support for clinical decision-making. Papers (37, 38) on surgical techniques and outcome evaluations, such as historical reviews of total hip arthroplasty and outcome analyses of reverse total shoulder arthroplasty, showcase continuous advancements in surgical techniques and ongoing optimization of treatment outcomes. In terms of quality of life and patient satisfaction, relevant papers (39, 40) delve into the impact of total hip and knee replacements on patients’ health-related quality of life, and emphasize the pivotal roles of pain management and functional recovery in patient satisfaction, underscoring the importance of postoperative rehabilitation and patient experience. In the field of epidemiology and prognosis research (41, 42), several papers reveal the current status of total hip and knee replacements, the disease burden of post-traumatic OA, and provide scientific bases for formulating public health policies and resource allocation. In addition, papers (43, 44) in the field of biomedical engineering and therapeutic technologies, such as discussions on articular cartilage repair and tissue engineering techniques, and long-term follow-up studies on metal-on-metal hybrid surface joint replacements, demonstrate the critical role of technological innovation in advancing arthritis treatment. In summary, these 30 highly cited papers play a pivotal role in academic research in the fields of arthritis and joint replacement surgery, driving progress in clinical practice and improving patients’ quality of life. Their profound insights and valuable data provide indispensable references and guidance for future research directions and clinical decisions.

Treatment for arthritis patients has made significant progress over the past few decades, historically ranging from anti-inflammatory drug therapy to joint replacement surgery. Joint replacement surgery can effectively alleviate pain and stiffness, reduce morbidity, and provide good to excellent function in medium- to long-term follow-ups (45). In the fields of surgical techniques and treatment outcomes for joint replacement surgery, Michael A Mont (46), Nicholas A Bellamy (47), and David T Felson (48), among others, have dedicated themselves to advancing orthopedic surgical techniques and improving patients' treatment outcomes through extensive research and clinical practice. From the analysis of keyword bursts, it is evident that, in addition to common terms such as RA, joint replacement surgery, and related anatomical areas, the prominence of keywords such as kinematic alignment, mechanical alignment, prostheses, PJI, and follow-up is also highly notable. These keywords indicate that current research is progressing toward deeper and more diversified directions, increasingly focusing on postoperative psychological states of patients, the recovery of joint functions, and the refinement of surgical techniques.

Especially in recent years, the highly trending keywords ‘kinematic alignment’ and ‘mechanical alignment’ underscore that mechanical surgical techniques and surgical precision have become hotspots and focal points of current research. Mechanical alignment and kinematic alignment are the two standard alignment techniques for implant placement in joint replacement surgery (49). Mechanical alignment is the traditional approach in TKA, aiming to restore the mechanical axis of the lower limb (i.e., the straight alignment of the hip, knee, and ankle) to achieve mechanical balance in the joint (50, 51). Early results of robotic-assisted joint replacement surgeries demonstrate potential benefits, including improved postoperative comfort, reproducibility, and a reduction in outliers (52). In recent years, robotic TKA, in comparison to manual TKA, has shown benefits such as reduced hospital stays, a higher probability of patients being discharged directly home, and a decreased chance of readmission within 90 days (53). Kinematic alignment restores the patient’s original anatomical joint structure, preserving its natural kinematic characteristics, which can lead to faster postoperative recovery and less pain. However, Kinematic alignment is not suitable for patients with significantly lower limb deformities or severe arthritis, as their original anatomical structures may be severely compromised. Additionally, compared to mechanical alignment, kinematic alignment places higher technical demands on surgeons (54, 55).

The outcome of joint replacement surgery is not only influenced by the method of prosthesis implantation but is also critically determined by the choice of prosthetic materials, which significantly impacts the long-term results of the procedure. As patients’ life expectancy increases and the proportion of younger patients undergoing surgery rises, the lifespan of prostheses and the demand for revision surgeries are becoming increasingly prominent (56). Currently, prostheses made from materials such as metal, polyethylene, and bioceramics are widely used in clinical practice, each offering unique advantages and limitations. For instance, bio-ceramic prostheses exhibit excellent wear resistance but carry the risk of fracture, while metal prostheses, despite their durability, may trigger complications such as metal ion release and local tissue reactions (57, 58). Although the success rate of joint replacement surgery is relatively high, postoperative complications such as infection, prosthetic loosening, dislocation, and deep vein thrombosis remain significant concerns. Among these, infection is one of the most severe complications (59, 60), potentially leading to surgical failure and a marked decline in patients’ quality of life.

PJI is a serious and challenging complication of joint replacement surgery. Significant advancements have been made in the diagnosis and treatment of PJI, including antibiotic therapy and a novel treatment known as continuous local antibiotic perfusion (61). Furthermore, through author analysis, we can observe that in the areas of long-term outcomes of joint replacement surgery, prosthesis durability, and postoperative complications, Stephen E Graves (62) and John N Insall (63) have conducted the most in-depth research and are recognized as authoritative experts in these fields.

With increasing demands for surgical precision, future research may further concentrate on optimizing personalized medical treatments and surgical strategies, including customized surgical approaches based on specific patient conditions, such as integrating kinematic or mechanical alignment technologies, to enhance long-term surgical outcomes and patient quality of life. Developing more wear-resistant and biocompatible materials, improving prosthetic design to reduce wear particle generation, exploring strategies to extend prosthesis lifespan and reduce revision rates, and optimizing techniques and methods for revision surgeries. In addition, research on postoperative management and rehabilitation strategies will remain crucial, particularly focusing on supporting and evaluating patients’ postoperative psychological states and promoting comprehensive recovery of joint functions. These efforts will provide crucial support for enhancing surgical outcomes and patients’ quality of life.

This study conducts a multi-level and multi-dimensional in-depth analysis of the literature related to joint replacement over the past few decades, revealing the evolutionary patterns of joint replacement surgery for arthritis patients and highlighting current research hotspots. However, several limitations should be acknowledged. First, while the WOSCC database provides the most comprehensive coverage of publications, some papers from other databases, including PubMed, Google Scholar, and Embase, were not extensively searched. Second, this study only analyzed English articles and reviews, excluding meeting abstracts, letters, case reports, editorials, book chapters, abstracts, and retractions, potentially causing selection bias. Third, due to the rapid growth and large volume of literature in this research field, the study had to make choices during data analysis and processing, focusing only on selected representative research results. Since citation metrics are time-dependent, this means that the newer high-quality literature may have fewer citations. Therefore, future research should explore more efficient methods for data processing and analysis to comprehensively capture and interpret all relevant information within the field.

## Conclusion

From 2004 to 2024, there has been a steady increase in the number of publications on joint replacement for treating arthritis. The US leads in publication volume, followed by the UK and China. Leading academic institutions such as Harvard University, the University of California system, and the Mayo Clinic demonstrate exceptional performance in both publication quantity and international collaboration. Promoting deeper cooperation and communication among international organizations and authors is crucial for accelerating the application of research findings. Academic journals explore theoretical discussions, share clinical practices, and highlight technological breakthroughs. Current research focuses on enhancing mechanical surgical techniques, improving surgical precision, and innovating post-operative management and rehabilitation strategies to offer safer and more effective treatment solutions.

## Supplementary materials



## ICMJE Statement of Interest

The authors declare that  that there is no conflict of interest that could be perceived as prejudicing the impartiality of the research reported.

## Funding Statement

The authors disclose receipt of the following financial or material support for the research, authorship, and/or publication of this article: this work was supported by the National Natural Science Foundation of Chinahttps://doi.org/10.13039/501100001809 (grant number 82273710).

## Author contribution statement

S-ST was responsible for conceptualization, writing the original draft, review and editing. J T contributed to data processing, chart creation, and writing review and editing. Y-CL contributed to methodology and writing review and editing. S-ZX participated in writing review and editing. H-FP and ZC critically reviewed the paper for intellectual content and final approval.

## Data availability

The datasets utilized in the current study are contained in the article.
