# Supplementary material for: Evolutionary patterns and future perspectives of joint replacement in arthritis patients: a comprehensive analysis of findings over the past decades
Source: EFORT Open Rev. 2025 Sep 4;10(9):646–59. doi: 10.1530/EOR-2025-0071 (PMC12412288; doi:10.1530/EOR-2025-0071)
Supplement: Supplementary file 1 [file Supplementary_materials.pdf]

Supplementary Table 1 The top 30 highly cited papers.

Rank	Article Title	First Author	Journal Title	Publication Year	Times Cited	Document Type	DOI
1	OARSI recommendations for the management of hip and knee osteoarthritis, Part II: OARSI evidence-based, expert consensus guidelines	Zhang, W et al(1)	Osteoarthritis and Cartilage	2008	1924	Review	10.1016/j.joca.2007.12.013
2	Osteoarthritis	Glyn-Jones, S et al(2)	Lancet	2015	1807	Article	10.1016/S0140-6736(14)60802-3
3	The operation of the century: total hip replacement	Learmonth, ID et al(3)	Lancet	2007	1589	Review	10.1016/S0140-6736(07)60457-7
4	Health-Related Quality of Life in Total Hip and Total Knee Arthroplasty A Qualitative and Systematic Review of the Literature	Ethgen, O et al(4)	Journal of Bone and Joint Surgery-American Volume	2004	1346	Review	10.2106/00004623-200405000-00012
5	Prevalence of Total Hip and Knee Replacement in the United States	Kremers, HM et al(5)	Journal of Bone and Joint Surgery-American Volume	2015	1058	Article	10.2106/JBJS.N.01141
6	Local clearance of senescent cells attenuates the development of post-traumatic osteoarthritis and creates a pro-regenerative environment	Jeon, OH et al(6)	Nature Medicine	2017	913	Article	10.1038/nm.4324
7	What proportion of patients report long-term pain after total hip or knee replacement for osteoarthritis? A systematic review of prospective studies in unselected patients	Beswick, AD et al(7)	BMJ Open	2012	902	Review	10.1136/bmjopen-2011-000435
8	Repair and tissue engineering techniques for	Makris, EA et al(8)	Nature Reviews	2015	866	Review	10.1038/nrrheum.

	articular cartilage		Rheumatology				2014.157
9	Atlas of individual radiographic features in osteoarthritis, revised	Altman, RD et al(9)	Osteoarthritis and Cartilage	2007	831	Article	10.1016/j.joca.2006.11.009
10	Fabry disease	Germain, DP et al(10)	Orphanet Journal of Rare Diseases	2010	801	Review	10.1186/1750-1172-5-30
11	Knee replacement	Carr, AJ et al(11)	Lancet	2012	773	Article	10.1016/S0140-6736(11)60752-6
12	Synovial tissue inflammation in early and late osteoarthritis	Benito, MJ et al(12)	Annals of the Rheumatic Diseases	2005	729	Article	10.1136/ard.2004.025270
13	Diagnosis and Treatment of Hip and Knee Osteoarthritis: A Review	Katz, JN et al(13)	JAMA-Journal of the American Medical Association	2021	723	Review	10.1001/jama.2020.22171
14	The role of pain and function in determining patient satisfaction after total knee replacement - Data from the National Joint Registry for England and Wales	Baker, PN et al(14)	The Journal of Bone and Joint Surgery-British volume	2007	675	Article	10.1302/0301-620X.89B7.19091
15	Reverse Total Shoulder Arthroplasty: A Review of Results According to Etiology	Wall, B et al(15)	Journal of Bone and Joint Surgery-American Volume	2007	675	Article	10.2106/JBJS.F.00666
16	Posttraumatic Osteoarthritis: A First Estimate of Incidence, Prevalence, and Burden of Disease	Brown, TD et al(16)	Journal of Orthopaedic Trauma	2006	650	Article	10.1097/01.bot.0000246468.80635.ef
17	Prevalence of knee osteoarthritis in the United States: Arthritis data from the Third National Health and Nutrition Examination Survey	Dillon, CF et al(17)	Journal of Rheumatology	2006	606	Article	PMID: 17013996

	1991-94						
18	EULAR evidence based recommendations for the management of hip osteoarthritis: report of a task force of the EULAR Standing Committee for International Clinical Studies Including Therapeutics (ESCISIT)	Zhang, W et al(18)	Annals of the Rheumatic Diseases	2005	600	Review	10.1136/ard.2004.028886
19	Increasing Incidence of Shoulder Arthroplasty in the United States	Kim, SH et al(19)	Journal of Bone and Joint Surgery-American Volume	2011	599	Article	10.2106/JBJS.J.01994
20	The reverse shoulder prosthesis for glenohumeral arthritis associated with severe rotator cuff deficiency - A minimum two-year follow-up study of sixty patients	Frankle, M et al(20)	Journal of Bone and Joint Surgery-American Volume	2005	594	Article	10.2106/JBJS.D.02813
21	Clinical practice. Infection associated with prosthetic joints	Del Pozo, JL et al(21)	The New England Journal of Medicine	2009	582	Article	10.1056/NEJMcp0905029
22	A systematic review of recommendations and guidelines for the management of osteoarthritis: The Chronic Osteoarthritis Management Initiative of the U.S. Bone and Joint Initiative	Nelson, AE et al(22)	Seminars in Arthritis and Rheumatism	2014	533	Review	10.1016/j.semarthrit.2013.11.012
23	Metal-on-metal resurfacing of the hip in patients under the age of 55 years with osteoarthritis	Daniel, J et al(23)	The Journal of Bone and Joint Surgery-British volume	2004	522	Article	10.1302/0301-620X.86B2.14600
24	Complications of total shoulder arthroplasty	Bohsali, KI et al(24)	The Journal of Bone and Joint	2006	501	Review	10.2106/JBJS.F.00125

			Surgery-British volume				
25	Comparison of the subjective shoulder value and the Constant score	Gilbart, MK et al(25)	Journal of Shoulder and Elbow Surgery	2007	491	Article	10.1016/j.jse.2007.02.123
26	OARSI recommended performance-based tests to assess physical function in people diagnosed with hip or knee osteoarthritis	Dobson, F et al(26)	Osteoarthritis and Cartilage	2013	483	Article	10.1016/j.joca.2013.05.002
27	All cause and disease specific mortality in patients with knee or hip osteoarthritis: population based cohort study	Nüesch, E et al(27)	BMJ-British Medical Journal	2011	482	Article	10.1136/bmj.d1165
28	Reverse Total Shoulder Arthroplasty Survivorship Analysis of Eighty Replacements Followed for Five to Ten Years	Guery, J et al(28)	Journal of Bone and Joint Surgery-American Volume	2006	472	Article	10.2106/JBJS.E.00851
29	Metal-on-Metal Hybrid Surface Arthroplasty: Two to Six-Year Follow-up Study	Amstutz, HC et al(29)	Journal of Bone and Joint Surgery-American Volume	2004	464	Article	PMID: 14711942
30	The effect of patient age at intervention on risk of implant revision after total replacement of the hip or knee: a population-based cohort study	Bayliss, LE et al(30)	Lancet	2017	460	Article	10.1016/S0140-6736(17)30059-4

References:

1. Zhang W, Moskowitz RW, Nuki G, Abramson S, Altman RD, Arden N, et al. OARSI recommendations for the management of hip and knee osteoarthritis, Part II: OARSI evidence-based, expert consensus guidelines. Osteoarthritis Cartilage. 2008;16(2):137-62.
2. Glyn-Jones S, Palmer AJ, Agricola R, Price AJ, Vincent TL, Weinans H, et al. Osteoarthritis. Lancet. 2015;386(9991):376-87.

3. Learmonth ID, Young C, Rorabeck C. The operation of the century: total hip replacement. Lancet. 2007;370(9597):1508-19.
4. Ethgen O, Bruyère O, Richy F, Dardennes C, Reginster JY. Health-related quality of life in total hip and total knee arthroplasty. A qualitative and systematic review of the literature. J Bone Joint Surg Am. 2004;86(5):963-74.
5. Maradit Kremers H, Larson DR, Crowson CS, Kremers WK, Washington RE, Steiner CA, et al. Prevalence of Total Hip and Knee Replacement in the United States. J Bone Joint Surg Am. 2015;97(17):1386-97.
6. Jeon OH, Kim C, Laberge RM, Demaria M, Rathod S, Vasserot AP, et al. Local clearance of senescent cells attenuates the development of post-traumatic osteoarthritis and creates a pro-regenerative environment. Nat Med. 2017;23(6):775-81.
7. Beswick AD, Wylde V, Gooberman-Hill R, Blom A, Dieppe P. What proportion of patients report long-term pain after total hip or knee replacement for osteoarthritis? A systematic review of prospective studies in unselected patients. BMJ Open. 2012;2(1):e000435.
8. Makris EA, Gomoll AH, Malizos KN, Hu JC, Athanasiou KA. Repair and tissue engineering techniques for articular cartilage. Nat Rev Rheumatol. 2015;11(1):21-34.
9. Altman RD, Gold GE. Atlas of individual radiographic features in osteoarthritis, revised. Osteoarthritis Cartilage. 2007;15 Suppl A:A1-56.
10. Germain DP. Fabry disease. Orphanet J Rare Dis. 2010;5:30.
11. Carr AJ, Robertsson O, Graves S, Price AJ, Arden NK, Judge A, et al. Knee replacement. Lancet. 2012;379(9823):1331-40.
12. Benito MJ, Veale DJ, FitzGerald O, van den Berg WB, Bresnihan B. Synovial tissue inflammation in early and late osteoarthritis. Ann Rheum Dis. 2005;64(9):1263-7.
13. Katz JN, Arant KR, Loeser RF. Diagnosis and Treatment of Hip and Knee Osteoarthritis: A Review. Jama. 2021;325(6):568-78.
14. Baker PN, van der Meulen JH, Lewsey J, Gregg PJ. The role of pain and function in determining patient satisfaction after total knee replacement. Data from the National Joint Registry for England and Wales. J Bone Joint Surg Br. 2007;89(7):893-900.
15. Wall B, Nové-Josserand L, O'Connor DP, Edwards TB, Walch G. Reverse total shoulder arthroplasty: a review of results according to etiology. J Bone Joint Surg Am. 2007;89(7):1476-85.
16. Brown TD, Johnston RC, Saltzman CL, Marsh JL, Buckwalter JA. Posttraumatic osteoarthritis: a first estimate of incidence, prevalence, and burden of disease. J Orthop Trauma. 2006;20(10):739-44.
17. Dillon CF, Rasch EK, Gu Q, Hirsch R. Prevalence of knee osteoarthritis in the United States: arthritis data from the Third National Health and Nutrition Examination Survey 1991-94. J Rheumatol. 2006;33(11):2271-9.
18. Zhang W, Doherty M, Arden N, Bannwarth B, Bijlsma J, Gunther KP, et al. EULAR evidence based recommendations for the management of hip osteoarthritis: report of a task force of the EULAR Standing Committee for International Clinical Studies Including Therapeutics (ESCISIT). Ann Rheum Dis. 2005;64(5):669-81.
19. Kim SH, Wise BL, Zhang Y, Szabo RM. Increasing incidence of shoulder arthroplasty in the United States. J Bone Joint Surg Am. 2011;93(24):2249-54.

20. Frankle M, Siegal S, Pupello D, Saleem A, Mighell M, Vasey M. The Reverse Shoulder Prosthesis for glenohumeral arthritis associated with severe rotator cuff deficiency. A minimum two-year follow-up study of sixty patients. J Bone Joint Surg Am. 2005;87(8):1697-705.
21. Del Pozo JL, Patel R. Clinical practice. Infection associated with prosthetic joints. N Engl J Med. 2009;361(8):787-94.
22. Nelson AE, Allen KD, Golightly YM, Goode AP, Jordan JM. A systematic review of recommendations and guidelines for the management of osteoarthritis: The chronic osteoarthritis management initiative of the U.S. bone and joint initiative. Semin Arthritis Rheum. 2014;43(6):701-12.
23. Daniel J, Pynsent PB, McMinn DJ. Metal-on-metal resurfacing of the hip in patients under the age of 55 years with osteoarthritis. J Bone Joint Surg Br. 2004;86(2):177-84.
24. Bohsali KI, Wirth MA, Rockwood CA, Jr. Complications of total shoulder arthroplasty. J Bone Joint Surg Am. 2006;88(10):2279-92.
25. Gilbart MK, Gerber C. Comparison of the subjective shoulder value and the Constant score. J Shoulder Elbow Surg. 2007;16(6):717-21.
26. Dobson F, Hinman RS, Roos EM, Abbott JH, Stratford P, Davis AM, et al. OARSI recommended performance-based tests to assess physical function in people diagnosed with hip or knee osteoarthritis. Osteoarthritis Cartilage. 2013;21(8):1042-52.
27. Nüesch E, Dieppe P, Reichenbach S, Williams S, Iff S, Jüni P. All cause and disease specific mortality in patients with knee or hip osteoarthritis: population based cohort study. Bmj. 2011;342:d1165.
28. Guery J, Favard L, Sirveaux F, Oudet D, Mole D, Walch G. Reverse total shoulder arthroplasty. Survivorship analysis of eighty replacements followed for five to ten years. J Bone Joint Surg Am. 2006;88(8):1742-7.
29. Amstutz HC, Beaulé PE, Dorey FJ, Le Duff MJ, Campbell PA, Gruen TA. Metal-on-metal hybrid surface arthroplasty: two to six-year follow-up study. J Bone Joint Surg Am. 2004;86(1):28-39.
30. Bayliss LE, Culliford D, Monk AP, Glyn-Jones S, Prieto-Alhambra D, Judge A, et al. The effect of patient age at intervention on risk of implant revision after total replacement of the hip or knee: a population-based cohort study. Lancet. 2017;389(10077):1424-30.
